# On the Distribution of Nerve Fibres

**Published:** 1868-04

**Authors:** Rufus King Browne


### ON THE DISTRIBUTION OF NERVE FIBRES.

BY PROF. RUFUS KING BROWNE, M. D.

No anatomical inquiry has a more commanding bearing upon pathology in all its aspects, than the character and arrangement of nerves. For we cannot understand the character of many of the most important, or the most ordinary diseases, until we have ascertained the true disposition of a nervous apparatus.

The arrangement of a nervous apparatus, has yet to be ascertained, for the utmost confusion and contradiction prevails respecting the exact relation of the nerve fibre to the tissues with which it is associated.

Some affirm that there are nerve cells, as in the brain, unconnected with fibres. Some affirm that the nerve itself may be influenced by a nerve (ganglionic) cell without, not directly connected with it, while others suppose the fibre affects a particular tissue by reason of its structural continuity with it. By some, nerves are supposed to end in free extremities, by others to lose themselves in other tissue. Some maintain that there is a "nerve force" either electrical or similar thereto, while others regard the supposed "nerve force" as another mode or form of force. Some imagine that muscular contraction cannot take place without some power or "force" communicated to it by a nerve, while others suppose that the nerve itself is only a conductor of this "force" transmitted from the brain.

From this it is evident that we have not yet ascertained the arrangement of a nerve mechanism, much less its character, considered physiologically. The extreme or "peripheral" distribution of nerve fibres may be easily studied in certain forms of connective tissue, but the arrrangement of the most minute nerve fibres is a matter of such great difficulty, that the observer is compelled to search with the utmost care for a tissue, the natural character of which may happen to be favorable for his investigation. There are few textures which possess so many advantages as the bladder of the frog, especially *Hyla. arborea*, for the study of the relations of nerve fibres, fine and coarse, to the involuntary muscles, and other textures of the organ. It is so thin and transparent, that it may literally be regarded as a natural dissection and thinning out of some of the most delicate tissues. Its unstriped muscular fibres are extremely fine, and are slightly separated from one another. In the thin membrane of which the walls of the frog's bladder are composed, we may follow out the distribution of nerves: 1st, to the unstriped muscular tissue; 2d, to the surface of the mucous membrane; 4th, to the vessels; and—5th, to the connective tissue.

The bundles of *coarse, or dark-bordered fibres*, at the posterior part of the bladder, divide and subdivide, spreading out in the form of a lax network, and may be traced to their ultimate distribution in the thin tissue of the bladder. Here and there, over a portion of the bladder where no dark-bordered fibres or bundles of coarse fibres can be discerned, a plexus of very fine fibres can be detected, from which bundles of fine fibres diverge in different directions. These latter result from the division and subdivision of dark-bordered fibres.

The muscular fibre cells of the bladder itself, and of the small arteries, are crossed sometimes in two or three places by very fine nerve fibres, and occasionally the nerve fibre runs *parallel* with the muscular fibre-cell for some distance. These nerve fibres are extremely fine, but they are not directly connected in structure with any part of the muscle-fibre. They cross the fibre either obliquely or at right angles, and continue onward.

In many instances ganglia are seen in connection with the nerves ramifying among the muscular fibre cells, encircling the vessels, but whether the large dark-bordered fibres seen in the nerve-trunks pass directly to their distribution in this fine plexus, or in the first place become connected with ganglion cells, it is difficult to decide with certainty. But, I have traced some of the large fibres directly from the trunk to their distribution.

In all cases, not only are nerve fibres distributed in considerable numbers upon the external surface of the artery, ramifying in the connective tissue, but they may be seen among the circular fibres of the arterial coat. These nerves invariably form networks with wide meshes.

I have always observed that the fibres, resulting from the subdivision of the dark-bordered fibres are of less diameter than the parent trunk, but the area of the section of two fibres is invariably much greater than that of the parent trunk.

Generally the fibres divide dichotomously, but frequently a dark-bordered fibre is seen to divide into as many as three or four divisions, and in muscles, five or six still dark-bordered fibres have been seen to result from one. The fine fibres do not exhibit the dark-bordered character. They appear pale and granular, and are often less than the $\frac{1}{50000}$ of an inch in diameter. Even these, *however, are bundles of several still finer fibres.* It is absolutely certain that these pale fibres are true nerve fibres, for I have traced them directly to the dark-bordered fibres, the two being continuous. I have found in the same nerve trunk fine and coarse dark-bordered fibres, the fine fibre running in the same transparent matrix (sheath), with the dark-bordered fibre. It is very probable that the fine fibre is a filament of the sympathetic.

From these observations and many others of a similar character, upon the diaphragm of the white mouse, and the mylo-hyoid of the little green-tree frog, it has been forced upon my comprehension, that the supposed distribution of nerve fibres, by *terminating* in tissue is erroneous; and that nerve fibres in every tissue form a network without terminus.

I have no hesitation in supposing that nerve fibres are distributed to different tissues on the same plan.

Some regard the *sensibility* of the dentine to be due to nerve fibres, which extend into, and are incorporated in the structure, being persuaded that the soft-solid masses of substance called "fibrils," are nerve fibres. But this I think is purely theoretical, for the continuity of these fibrils with the mass of connective tissue in the pulp cavity, has been and can readily be demonstrated. Moreover, in all these histological characters, the fibrils are strikingly unlike nerve fibres. But, more than this, if the fibrils are truly the seat of sensibility of the dentinal tissue, they would be found structurally connected with the nerves of the pulp. But these clusters of nerves possess all the ordinary and distinguishing character of the nerve fibres.

Some believe that sensibility exists without nerve tissue, but if this be true, it does not support the idea that in situations where nerve tissue really exists, as it does in the tooth, another kind of tissue, different from it, as the dentinal fibrils are, is a sensitive tissue.

---

[NOTE.—The claim that nerve fibres never terminate or come to an end, in tissue, was first confidently announced by the distinguished English microscopist Dr. Lionel S. Beale, of London. On the other hand, the continental observers almost unanimously affirm, that both motor and sensitive nerves end in the tissues. Van der Kolk, claiming that wherever a mixed (motor and sensitive) branch runs to a part distinct from the brain, the sensitive fibres terminate in or on the surface of that part.]

